# Structural and Photoconductivity Properties of Tellurium/PMMA Films

**DOI:** 10.1186/s11671-015-1007-z

**Published:** 2015-08-06

**Authors:** Gianfranco Carotenuto, Mariano Palomba, Sergio De Nicola, Giuseppina Ambrosone, Ubaldo Coscia

**Affiliations:** Institute for Polymers, Composites and Biomaterials, National Research Council, Piazzale E. Fermi 1, 80055 Portici, Naples Italy; SPIN Institute, National Research Council, via Cintia, 80126 Naples, Italy; Department of Physics, University of Naples ‘Federico II’, via Cintia, 80126 Naples, Italy; CNISM, Naples Unit, via Cintia, 80126 Naples, Italy

**Keywords:** Tellurium, Vibration milling, Poly(methyl methacrylate), Photoconductivity

## Abstract

Owing to the very brittle nature of tellurium powder, nanoscopic grains with an average size of 4.8 ± 0.8 nm were produced by dry vibration milling technique using a mixer/mill apparatus. A novel material was obtained by binding the nanosized tellurium grains with poly(methyl methacrylate) (PMMA) polymer. The morphology, elemental composition, and structural and optical properties of Te/PMMA films were investigated. The prepared material was composed of hexagonal tellurium and α-phase of tellurium oxide. The electrical properties of the films were studied, for different electrode contact configurations, in dark condition and under white light illumination varying the optical power density from 2 to 170 mW/cm^2^ and turning the light on and off cyclically. Data analysis shows that the photoconductivity of the film with sandwich contact configuration is a linear function of the light power density and increases more than 2 orders of magnitude as compared to the photoresponse of the film with coplanar contact configuration.

## Background

Elemental tellurium is a p-type semiconductor that can be exploited for many technological applications in metallurgy, photovoltaics, photonics, electronics, and medicine [1]. It has been used in the form of thin films or powder to fabricate gas sensors [2, 3], antiseptic materials [4], photoconductors [5–7], thermoelectric devices [8–11], etc. Usually, chemical and electrochemical methods (“bottom-up” approaches), such as chemical vapor deposition [12] and solvothermal synthesis [13], are utilized to produce tellurium-based materials. In particular, one-dimensional (1D) tellurium nanostructures such as wires, rods, tubes, and belts have been synthesized. For example, Rao et al. [14] reported controlled synthesis of Te nanorods, nanowires, nanobelts, and related structures by the disproportionation of NaHTe in different solvents, Xia et al. [15, 16] prepared uniform Te nanowires and nanotubes through the reduction of H_6_TeO_6_ by N_2_H_4_H_2_O or ethylene glycol in refluxing process, and Qian’s group produced a series of 1D Te nanostructures including nanowires, nanobelts, and nanotubes via hydrothermal synthesis [17–20]. Recently, Zhu et al. [21] presented an ultrasonic-assisted solution-phase approach for the fabrication of tellurium bundles of nanowhiskers, Sen et al. [22] synthesized Te nanostructures by physical vapor deposition, and Vasileiadis et al. demonstrated that a controlled fabrication of Te nanotubes can be carried out by irradiating bulk elemental Te with continuous wave lasers emitting in visible range for short exposure time [23].

In this paper, results on a top-down approach [24], based on dry vibration milling technology, to reduce the size of a brittle material such as tellurium and produce nanoscopic phases in a simple, effective, and inexpensive way, are reported*.* Indeed, nanostructures in the form of fine tellurium powder composed of grains with average size of a few nanometers were produced in air, without any temperature control and chemical reactions.

Furthermore, a novel functional material was obtained as monolithic film by binding the tellurium nanopowders with poly(methyl methacrylate) (PMMA), an amorphous thermoplastic polymer widely used in spectroscopic and optoelectronic applications.

Further advantages of this preparation method are the following: (i) the slight toxicity of tellurium is reduced by embedding it in the form of powder into a polymeric matrix thus overcoming the limits for an industrial use, (ii) the tellurium in a hyperfine form allows to achieve high homogeneous tellurium-polymer composites suitable for optical and flexible electronic applications, and (iii) the structural, optical, and electrical properties of the tellurium nanocomposites can be tuned by reducing the tellurium size at nanoscopic scale (tellurium quantum dots).

In order to obtain good electrical transport properties of this tellurium-based material, structures with a filling factor higher than 30 % by weight for nanoscopic tellurium [25] were prepared. The morphology, elemental composition, structural and optical properties of tellurium/PMMA films were analyzed by scanning electron microscopy (SEM), energy-dispersive spectroscopy (EDS), transmission electron microscopy (TEM), X-ray diffraction (XRD), Fourier transform infrared (FT-IR), and UV-vis-NIR spectroscopies. The electrical properties were investigated in dark condition and under white light illumination with coplanar and sandwich electrode configurations. The time-dependent photocurrent responses were measured turning the light on and off cyclically at different optical power densities of the light.

## Methods

Pure tellurium powder (99.8 % by weight, −200 mesh, Aldrich) was placed inside a steel grinding jar with two steel grinding balls. The tellurium grains were dry milled in air at a frequency of 25 Hz, for 7 h using a Mixer Mill apparatus (Retsch, MM-200). The grinding jar performs oscillations in a horizontal position, and the balls impact with high energy on the material thus pulverizing it. The movement of the grinding jar combined with the movement of the balls results in the intensive frictional action on the Te powder. At a frequency of 25 Hz, thousands of impacts per minute are achieved, resulting in a high degree of tellurium pulverization in a very short time.

The obtained powders were converted into monolithic samples by using a little amount of poly(methyl methacrylate) (*M*_w_ = 996,000 g mol^−1^) as binder to fabricate Te/PMMA films of large area (ca. 20 cm^2^). In more detail, PMMA was dissolved in acetone at room temperature, then the powder was added and accurately dispersed by using a sonication bath, and finally, the liquid systems were spin-coated (60 min at 200 rpm.) on a silicon plate as substrate. The sample composition was 11 % by weight in polymer in order to reach a compromise between the minimum amount of polymer to bind the tellurium grains and the maximum concentration of tellurium to obtain suitable electrical properties for device applications. The thickness of the prepared Te/PMMA film, measured by a Millitron electrical length measuring instrument, was about 80 μm. The large-area film was cut into several specimens for each characterization.

The morphology of the tellurium powders and Te/PMMA films was investigated by SEM measurements performed by a FEI QUANTA 200 FEG apparatus equipped with an EDS microanalyzer (Inca Oxford 250), while nanoscopic grain size was determined by TEM measurements carried out by a FEI Tecnai G2 Spirit twin apparatus. In this case, the milled powder was dispersed into an amorphous polymeric matrix (polystyrene, Aldrich), using chloroform as solvent (solvent-mediated method) and depositing such tellurium/polystyrene system on the TEM copper grids. The structural characterizations of the powder samples and Te/PMMA films were carried out by X-ray diffraction and Fourier transform infrared spectroscopy using a PANalytical-X’Pert Pro diffractometer and Nicolet Nexus spectrophotometer, respectively.

The electrical properties were studied at room temperature in a coplanar configuration by depositing Ag paint contacts 4 mm long spaced by 1 mm and in a sandwich configuration covering the top and the bottom surfaces of the film with 3 and 10 mm^2^ of Ag contacts, respectively. The electrical measurements of the samples were performed in both contact configurations by a Keithley 6485 picoammeter and a Tektronics PS 280 DC power supply. Time-dependent current of the Te/PMMA films was measured switching on and off the white light illumination of an ELC 250 W lamp of General Electric. Optical power density of the light flux was varied from 2 to 170 mW/cm^2^ by means of neutral density filters and measured by a Laser Precision Rk-5720 power radiometer.

## Results and Discussion

### Structural and Morphological Analysis

**Fig. 1:**
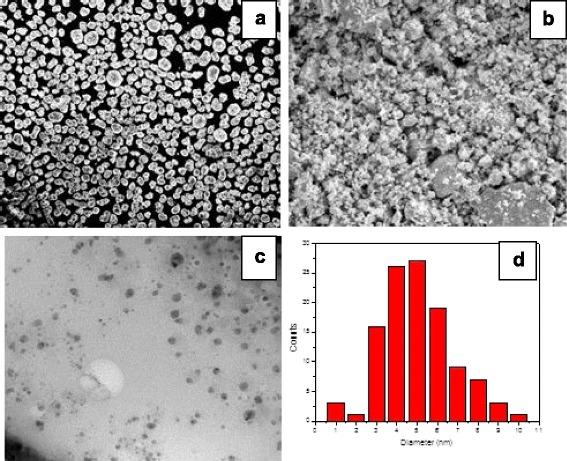
SEM micrographs of the “as received” tellurium powder (**a**) and the tellurium powder after milling (**b**). TEM-micrograph of the achieved nanoscopic tellurium grains embedded into an amorphous polystyrene matrix (**c**). Tellurium grain size distribution (**d**)

SEM and TEM measurements on Te powder samples were carried out to verify the ability of dry vibration milling technique to produce nanoscopic Te powder for the fabrication of Te/PMMA films. The “as received” tellurium powder was made of quite monodispersed pseudospherical grains with an average size of ca. 30 μm as visible in the SEM micrograph given in Fig. 1a, whereas the milled powder was characterized by a polymodal particle size distribution as displayed in the SEM micrograph in Fig. 1b. Most part of the powder was made of nanoscopic tellurium grains, as can be seen from the TEM image in Fig. 1c. The grain size distribution is shown in the histograms in Fig. 1d, and the average grain diameter was estimated to be 4.8 ± 0.8 nm.

**Fig. 2:**
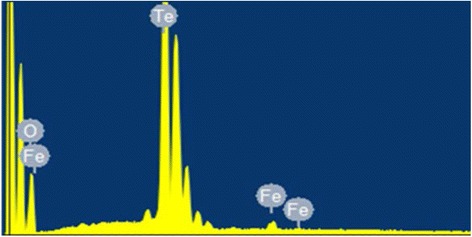
EDS spectrum of a milled tellurium powder sample

The composition of the milled powder was determined by EDS investigation (see Fig. 2). Data analysis indicates the presence of oxygen, due to the dry-milling process performed in air that can lead to a partial oxidation of the tellurium grain surface, and traces of iron, which is already present in the starting coarse tellurium powder.

**Fig. 3:**
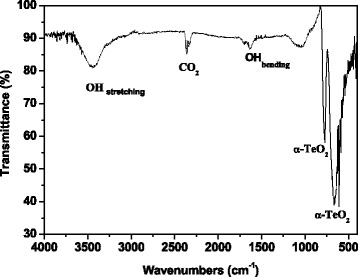
FT-IR spectrum of a milled tellurium powder sample

Some information on the structural properties of the milled powder was obtained by infrared spectroscopy. Indeed, the FT-IR spectrum of the milled tellurium sample shown in Fig. 3 includes the typical absorption bands of tellurium oxide centered at wavenumbers of 773 and 667 cm^−1^. According to the literature [26], these two resonances correspond to the symmetrical equatorial and asymmetrical axial stretching frequencies of the Te–O bonds, respectively. Furthermore, a quite broad and intensive absorption band due to the OH stretching vibrational mode, located at a wavenumber of 3436 cm^−1^, can be detected revealing that the oxide phase was partially hydrated.

Te/PMMA film of large area was obtained by binding the nanoscopic Te grains of the milled powder with PMMA.

**Fig. 4:**
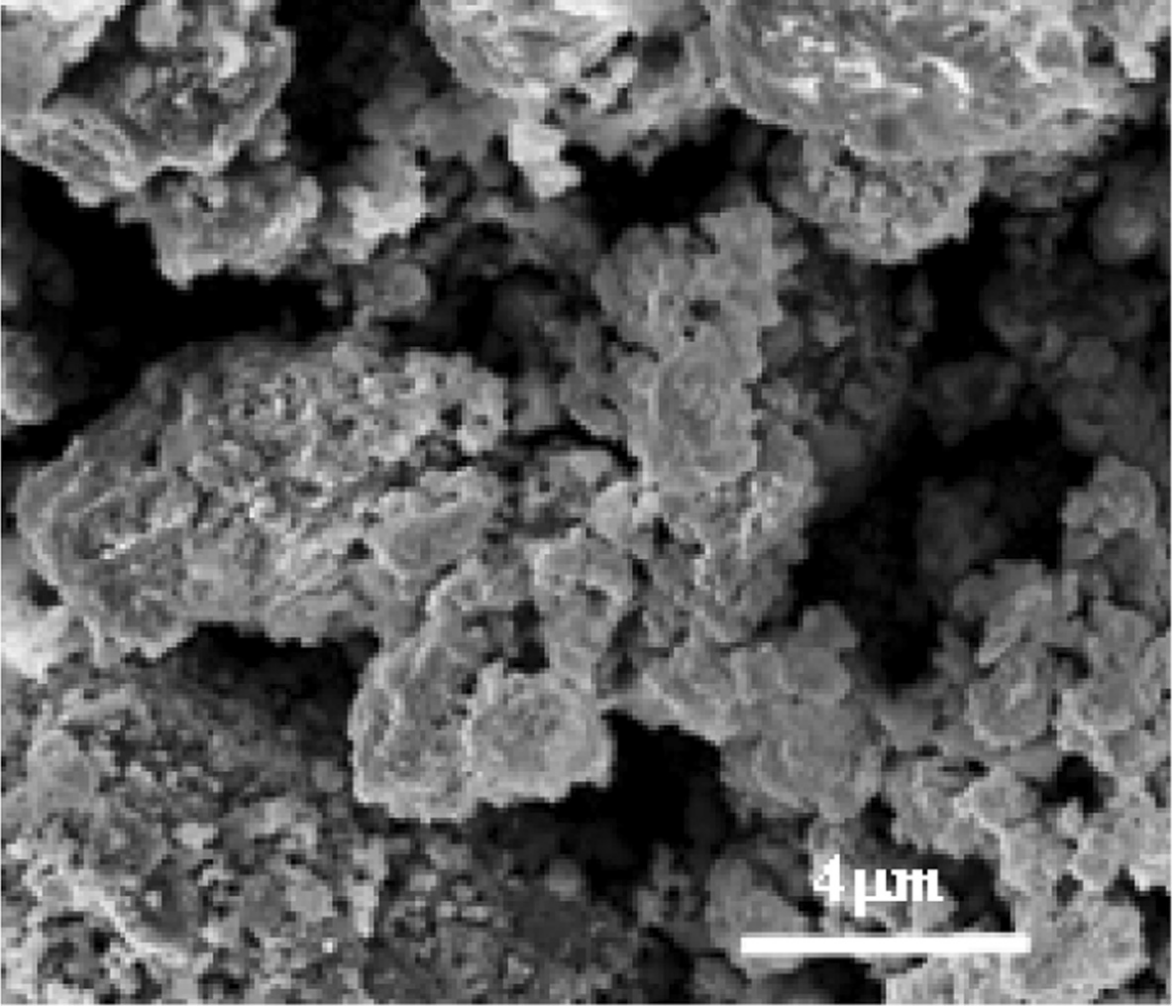
SEM micrograph of the Te/PMMA film

The morphology of the film was investigated by SEM. The micrograph, shown in Fig. 4, reveals that the material was quite porous, due to the very high inorganic phase content. In particular, both macro- and micro-porosities can be clearly observed as black areas in the image.

**Fig. 5:**
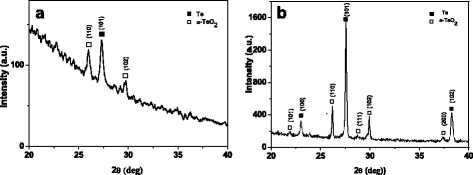
XRD diffractogram of the Te/PMMA film (**a**) and “as received” tellurium powder (**b**)

The structural properties of the Te/PMMA material were studied by XRD measurements. In Fig. 5a, the XRD diffractogram shows a prominent peak at 27.32° belonging to the diffraction pattern of the hexagonal tellurium (JCPDS card 36-1452) and two other less intensive diffraction peaks at 25.99° and 29.79° due to the α-phase of tellurium oxide (α-TeO_2_, JCPDS card 78-1713). Both Te and α-TeO_2_ phases were already present in the “as received” Te powder, as shown in the XRD spectrum of Fig. 5b; however, in the case of the Te/PMMA film, the peaks are broadened because the sample was composed of nanoscopic Te grains, achieved at the end of the milling process. Furthermore, according to the integrated area of the diffraction peaks, the composition of the film was 53 % Te and 47 % α-TeO_2_, while for the “as received” powder it was 80.8 % Te and 19.2 % α-TeO_2_. The higher value of the α-TeO_2_ phase percentage in the film can be ascribed to an oxidation process of the Te grains occurring during the milling stage.

### Optical and Photoconductivity Properties

**Fig. 6:**
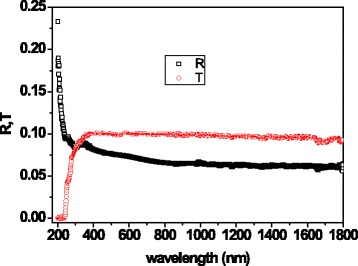
Transmittance, *T*, and reflectance, *R*, spectra of the Te/PMMA film in the UV-vis-NIR region

The optical properties of the Te/PMMA film were investigated by means of transmittance (*T*) and reflectance (*R*) spectroscopy in the UV-vis-NIR range. The *R*/*T* spectra of the film are shown in Fig. 6. The transmittance increases in the 200–350 nm range, and it is quite constant in the Vis and NIR ranges. The reflectance rapidly decreases in the UV region, slightly decreases in the Vis region, and is quite constant in the NIR region. Thus, from quantitative analysis, the sample absorptance varies in the 0.8–0.9 range in all the UV-vis-NIR regions.

**Fig. 7:**
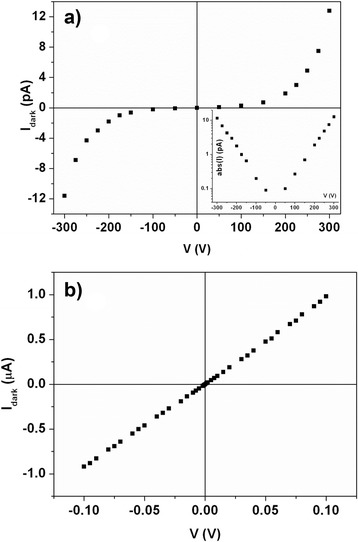
*I*–*V* characteristics of the Te/PMMA films in coplanar configuration (**a**) and sandwich configuration (**b**). In the inset, the absolute value of *I*_dark_, abs (I), as a function of the applied voltage *V* is plotted in semi-log scale

The electrical characterization of the Te/PMMA samples was performed both in coplanar and sandwich configurations. In the dark condition, the *I*–*V* characteristic in the coplanar configuration is nonlinear (see Fig. 7a) and the behavior of the absolute value of the current as a function of the applied voltage *V* is exponential as shown in the semilogarithmic plot of the inset in Fig. 7a. In the sandwich configuration, the *I*–*V* characteristic, displayed in Fig. 7b, is quite linear indicating that the contacts are ohmic.

The very different dark conductivity values obtained for the two configurations can be attributed to the film morphology as shown by the SEM image in Fig. 4.

**Fig. 8:**
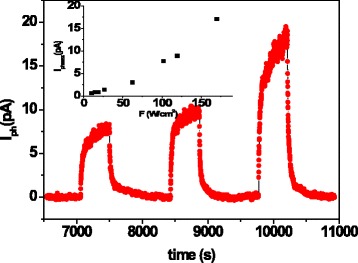
Time-dependent photocurrent, *I*_ph_, measurements for different light-dark cycles at various light power densities (100, 120, and 170 mW/cm^2^) for coplanar configuration. In the *inset*, the maximum photocurrent obtained for each cycle, *I*_phmax_, versus light power density, *F*, is displayed

In order to investigate the photoconductivity properties, the samples were illuminated by white light of different power density values. The current under illumination, *I*_light_, was measured in coplanar configuration, by applying a bias voltage of 200 V to reach a good signal-to-noise ratio avoiding high power dissipation. The illumination exposure time was held fixed at 440 s and immediately after the light was switched off to obtain light-dark cycles and evaluate the photocurrent, *I*_ph_, as *I*_ph_ = *I*_light_ − *I*_dark_, where *I*_dark_ was the dark current measured before turning on the light. The evolution of *I*_ph_ as a function of time for light-dark cycles performed by light power densities of 100, 120, and 170 mW/cm^2^ is shown in Fig. 8. The photocurrent signal slowly increases under illumination, and the rise time varies in the 100–300 s range. Upon turning off the light, the signal decreases slowly and the decay time varies in the same range*.* The inset in Fig. 8 shows the maximum value of the photocurrent for each cycle, *I*_phmax_, as a function of power density *F* in the 10–170 mW/cm^2^ range.

**Fig. 9:**
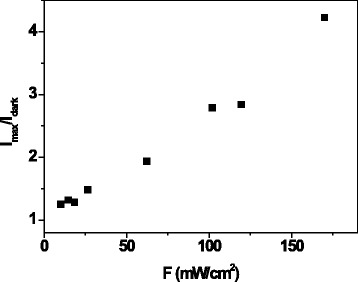
*I*_max_/*I*_dark_ ratio (where *I*_max_ is the maximum current obtained for each light-dark cycle and *I*_dark_ is the current in dark condition) versus light power density *F* for Te/PMMA film in coplanar configuration

In order to compare the photoconductivity measurements with the literature data concerning the coplanar configuration, the *I*_max_/*I*_dark_ ratio versus the light power density *F* is plotted in Fig. 9, where *I*_max_ = *I*_phmax_ + *I*_dark_ is the maximum value of the current before turning off the light. The *I*_max_/*I*_dark_ ratio depends quasilinearly on *F*, and it is worth noting that *I*_max_/*I*_dark_ = 2.8 at *F* = 100 mW/cm^2^; thus, the Te/PMMA sample shows a good photosensitivity as in the case of films composed of tellurium nanorods immersed in polydimethylsiloxane [27].

**Fig. 10:**
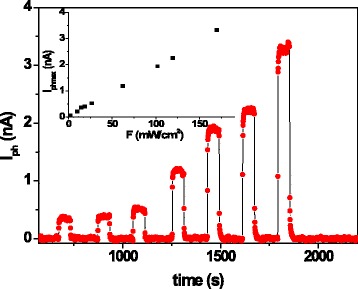
Time-dependent photocurrent *I*_ph_ measurements for different light-dark cycles with light power density *F* ranging from 15 to 170 mW/cm^2^ for sandwich configuration. In the *inset*, the maximum photocurrent obtained for each cycle, *I*_phmax_, is plotted as a function of light power density *F*

The photoresponse of the Te/PMMA sample in the sandwich configuration for different light-dark cycles is shown in Fig. 10. The measurements were carried out by applying a bias voltage of 12 μV to the sample varying the light power density from 2 to 170 mW/cm^2^. The duration of the illumination exposure was fixed at 60 s for each cycle. In this case, the rise time of the signal was about 4 s. When the light is removed, the current quickly decreases with a decay time of about 2 s. The inset in Fig. 10 shows that *I*_phmax_ depends linearly on *F*.

According to refs. [6, 23, 28], the origin of photoconduction in this material may be attributed to the coexistence of Te and TeO_2_ phases due to the partial oxidation of Te grains as demonstrated by XRD analysis.

**Fig. 11:**
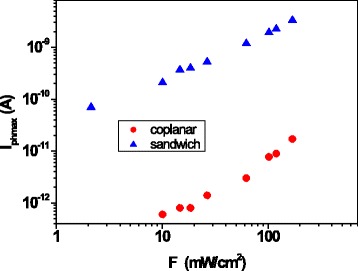
The maximum photocurrent obtained for each light-dark cycle, *I*_phmax_, versus light power density, *F*, for sandwich and coplanar configurations

In order to compare the photoresponse of the samples for the two contact configurations, the photocurrent *I*_phmax_ is plotted as a function of *F* in Fig. 11 in bi-logarithmic scale. Clearly, in the case of the sandwich configuration, the photocurrent obtained applying only tens of microvolts of bias voltage is more than 2 orders of magnitude greater than the one measured in the coplanar configuration by applying 200 V.

## Conclusions

It has been demonstrated that dry vibration milling is a suitable technology for producing tellurium nanoscopic powders. A novel material based on nanosized tellurium bound by means of a thermoplastic polymer such as PMMA was prepared.

The morphological characterizations of Te powders and Te/PMMA films were performed by SEM and TEM, and the films showed a quite porous nature due to the low amount of polymer present in the film. From XRD data analysis, it was obtained that the Te/PMMA material was composed of hexagonal Te and α-phase of tellurium oxide. The optical absorptance of the Te/PMMA sample was found varying in the 0.8–0.9 range in the UV-vis-NIR region. The time-dependent photoconductivity properties of Te/PMMA films were explored under white light illumination, turning the light on and off cyclically in coplanar and sandwich contact configurations. The photoresponse was studied as a function of the optical power density in the 2–170 mW/cm^2^ range. In the coplanar configuration, the rise and decay times of the photocurrent signal are on the order of hundreds of seconds, while in the sandwich configuration, the signal varies faster and the rise and decay times are just a few seconds. Finally, for the sandwich configuration, a linear correlation between the photocurrent and optical power density, and higher values of the photoresponse were found.
